# Anti-CD38 monoclonal antibodies in multiple myeloma and beyond: immunotherapeutic mechanisms, evidence maturity, and clinical positioning

**DOI:** 10.3389/fimmu.2026.1932951

**Published:** 2026-09-01

**Authors:** Liming Yu, Changnian Li, Dadong Guo, Siyuan Cui

**Affiliations:** 1 Shandong University of Traditional Chinese Medicine, First Clinical Medical College, Jinan, China; 2 Shandong First Medical University Third Affiliated Hospital, Jinan, China; 3 Shandong Key Laboratory of Integrated Traditional Chinese and Western Medicine for Prevention and Therapy of Ocular Diseases; Medical College of Optometry and Ophthalmology, Shandong University of Traditional Chinese Medicine, Jinan, China; 4 Shandong Academy of Eye Disease Prevention and Therapy, Jinan, China; 5 Affiliated Hospital of Shandong University of Traditional Chinese Medicine, Institute of Hematology, Shandong University of Traditional Chinese Medicine, Jinan, China

**Keywords:** anti-CD38 monoclonal antibodies, cancer immunotherapy, daratumumab, humoral immunity, isatuximab, minimal residual disease, multiple myeloma, resistance

## Abstract

Anti-CD38 monoclonal antibodies have become a central immunotherapeutic platform in multiple myeloma, where they have reshaped treatment across relapsed or refractory disease, frontline therapy, maintenance intensification, and minimal residual disease-oriented management. Their success in multiple myeloma has also stimulated broader development in immunoglobulin light-chain amyloidosis, selected hematologic malignancies, and humoral immune-mediated disorders. CD38 functions both as a surface target and as an ectoenzyme involved in nicotinamide adenine dinucleotide metabolism, calcium signaling, immune-cell regulation, and microenvironmental remodeling, providing a mechanistic basis for antibody-mediated cytotoxicity, immune modulation, and broader humoral immune intervention. This Review critically evaluates CD38 biology, Fc-mediated immune-effector mechanisms, immune remodeling, clinical evidence, resistance, safety, laboratory interferences, biomarker evidence, and clinical positioning of anti-CD38 antibodies, with multiple myeloma as the central reference disease. In immunoglobulin light-chain amyloidosis, daratumumab-based therapy has moved beyond myeloma-derived extrapolation and established a disease-specific framework integrating hematologic response, organ response, and survival outcomes. Beyond multiple myeloma, anti-CD38-based strategies have been investigated in light-chain amyloidosis, selected hematologic malignancies, autoimmune cytopenias, transplant-related alloimmunity, and other antibody-driven diseases. However, the strength of evidence differs substantially across these settings, and CD38 expression alone does not establish clinical benefit. Outside established plasma cell disorders, further development should therefore rely on disease-specific prospective evidence and careful assessment of safety.

## 1 Background

Anti-CD38 monoclonal antibodies have become an important therapeutic drug class in plasma cell disorders and are now being explored in selected humoral immune-mediated diseases. In multiple myeloma (MM), despite major advances achieved with proteasome inhibitors, immunomodulatory agents, and CD38-targeting monoclonal antibodies, relapse remains common, and improving the depth, durability, and clinical translation of response remains a central therapeutic goal (1, 2). The clinical importance of this drug class extends beyond direct plasma-cell depletion. CD38 is both a surface target and a multifunctional ectoenzyme involved in nicotinamide adenine dinucleotide metabolism, calcium signaling, immune-cell activation, and microenvironmental regulation (3). Accordingly, anti-CD38 therapy should be understood not only as a cytotoxic strategy against CD38-expressing cells, but also as a pharmacologic platform integrating plasma-cell depletion, Fc-mediated immune effector mechanisms, and broader modulation of humoral immune dysregulation.

**Figure 1:**
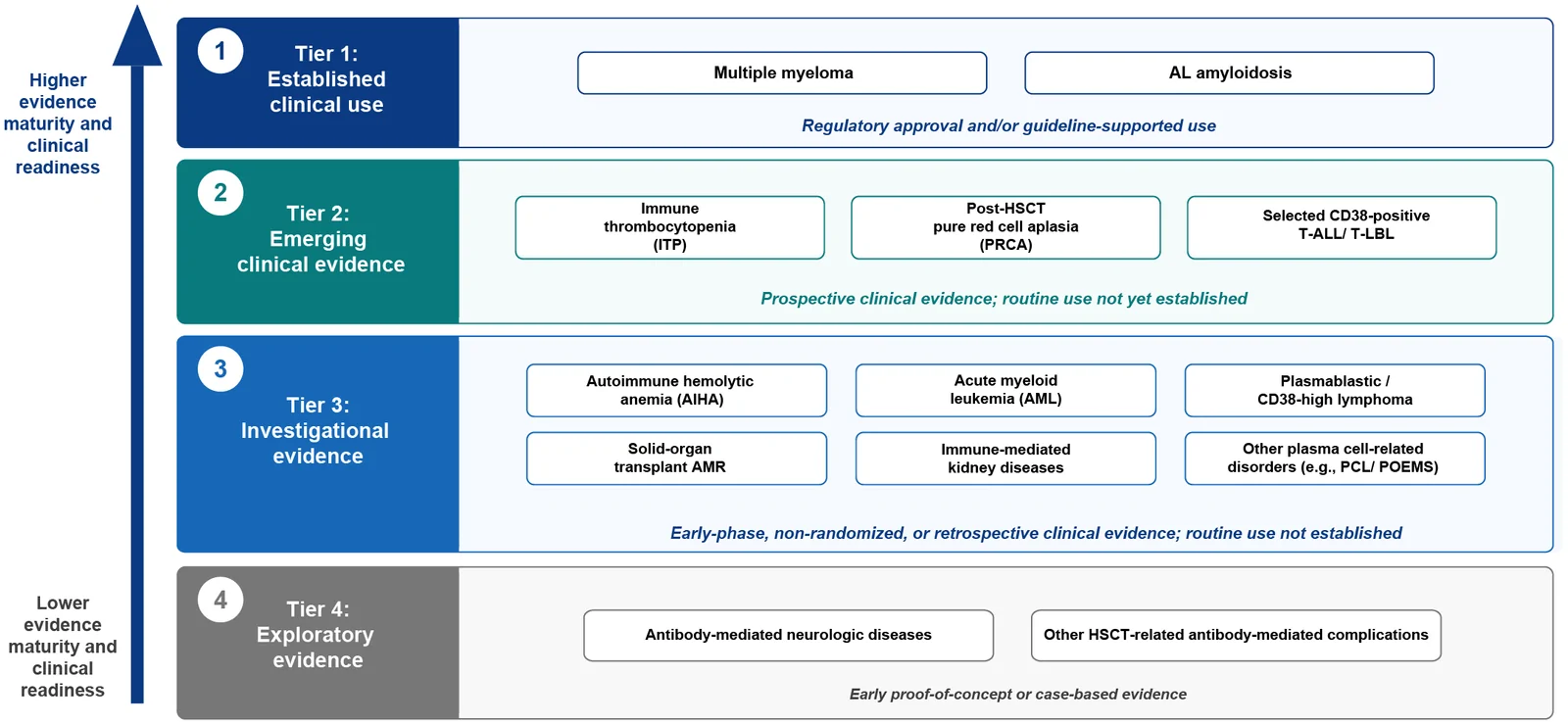
Evidence maturity and clinical readiness of anti-CD38 monoclonal antibodies across established and emerging indications. Indications were positioned according to the overall maturity of clinical evidence, considering study design, sample size in the context of disease prevalence, maturity of follow-up, clinically relevant endpoints, consistency across independent studies, and current regulatory or guideline-supported status. Tier 1 represents established clinical use supported by regulatory approval and/or guideline-supported practice together with mature prospective evidence. Tier 2 includes indications with substantive prospective clinical evidence but without established routine use. Tier 3 includes investigational indications supported mainly by early-phase, non-randomized, or retrospective clinical evidence without sufficient independent validation. Tier 4 represents exploratory applications supported predominantly by case-based or mechanistic evidence. This framework summarizes evidence maturity and clinical readiness and should not be interpreted as formal treatment recommendations. Representative indications are shown, and the framework is not intended to be exhaustive.

The clinical development of this drug class is most mature in MM and immunoglobulin light-chain amyloidosis. In MM, daratumumab- and isatuximab-based regimens have moved from relapsed or refractory disease into frontline therapy, post-transplant strategies, and minimal residual disease-oriented treatment approaches (4–6). In light-chain amyloidosis, daratumumab-based therapy has established a disease-specific framework in which rapid hematologic response, organ response, and survival benefit are integrated into therapeutic assessment (7, 8). More recently, CD38-targeted approaches have been explored in selected non-plasma cell hematologic malignancies (9), autoimmune diseases (10) and transplant-related immune responses (11). However, these indications differ substantially in target biology, immune-effector context, evidence maturity, clinical readiness, and safety considerations. Therefore, the expanding use of anti-CD38 therapy creates a need for critical appraisal rather than broad extrapolation from CD38 expression alone. This Review evaluates the biological rationale, clinical evidence, safety issues, laboratory interferences, resistance mechanisms, and disease-specific positioning of anti-CD38 monoclonal antibodies, with an emphasis on distinguishing established standards from promising but still investigational or exploratory applications. A conceptual framework for evidence-based positioning of anti-CD38 monoclonal antibodies across established, emerging, investigational, and exploratory indications is shown in **Figure 1**.

Relevant literature was identified through searches of PubMed/MEDLINE and Web of Science Core Collection from database inception through August 1, 2026. Search terms included combinations of “CD38”, “anti-CD38”, “daratumumab”, “isatuximab”, “felzartamab”, “mezagitamab”, and “CM313” with relevant disease- and topic-specific terms, including “multiple myeloma”, “amyloidosis”, “leukemia”, “lymphoma”, “immune thrombocytopenia”, “autoimmune hemolytic anemia”, “autoimmune disease”, “kidney disease”, “transplantation”, “minimal residual disease”, “resistance”, “safety”, and “biomarker”. Randomized trials, prospective studies, pivotal trials, and mature follow-up analyses were prioritized. For uncommon or emerging indications with limited prospective evidence, retrospective cohorts and case series were considered, while individual case reports were included when they provided clinically informative evidence not represented in larger studies. Reference lists of relevant reviews and primary studies were also screened. Evidence was interpreted according to study design, sample size, follow-up maturity, clinically relevant endpoints, independent validation, and current clinical or regulatory status; regulatory approval, guideline-supported practice, prospective investigational evidence, retrospective evidence, and off-label or experimental use were distinguished where applicable.

## 2 CD38 biology and immunotherapeutic mechanisms in multiple myeloma and related immune disorders

**Figure 2:**
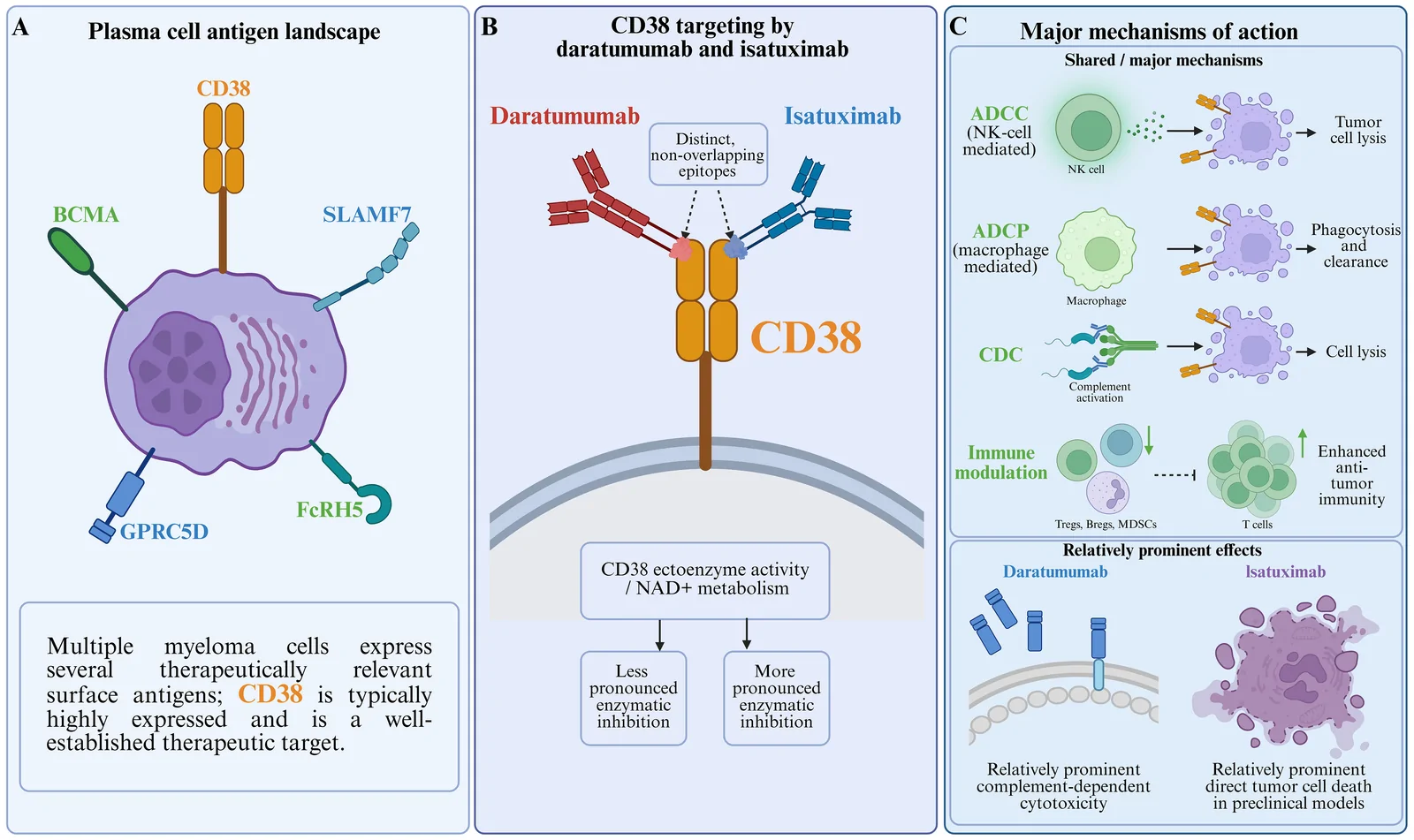
Plasma cell antigen landscape and major mechanisms of action of daratumumab and isatuximab. **(A)** Multiple myeloma cells express several therapeutically relevant surface antigens, including CD38, BCMA, SLAMF7, GPRC5D, and FcRH5. CD38 is typically highly expressed and represents a well-established therapeutic target in multiple myeloma. **(B)** Daratumumab and isatuximab bind distinct, non-overlapping epitopes of CD38. Both antibodies target CD38 as a surface antigen but differ in their effects on CD38 ectoenzyme activity, with enzymatic inhibition being more pronounced with isatuximab than with daratumumab. **(C)** Major shared mechanisms of anti-CD38 activity include antibody-dependent cellular cytotoxicity, antibody-dependent cellular phagocytosis, complement-dependent cytotoxicity, and immune modulation. The latter includes reduction of CD38-positive immunoregulatory cell populations and expansion of T cells, contributing to enhanced antitumor immunity. Some mechanisms may be relatively more prominent for individual antibodies: complement-dependent cytotoxicity is particularly prominent with daratumumab, whereas direct tumor-cell death has been demonstrated more prominently with isatuximab in selected preclinical models. These differences are relative rather than exclusive, and the contribution of individual mechanisms may vary according to antibody, CD38 expression, effector-cell context, and experimental conditions.

CD38 is an attractive therapeutic target because it combines surface accessibility with biologically relevant enzymatic and immunologic functions. CD38 expression is dynamically regulated during B-cell development rather than being restricted to the plasma-cell stage. Early B-lineage precursors in the bone marrow express CD38, whereas its expression decreases as cells mature into resting peripheral B-cell populations. CD38 is subsequently re-expressed during B-cell activation and terminal differentiation, with particularly high surface expression on plasmablasts and plasma cells (12, 13). This differentiation-associated pattern is therapeutically relevant because malignant plasma cells retain high CD38 expression, providing a direct rationale for antibody-mediated depletion in multiple myeloma. It also helps explain the broader interest in CD38 targeting in humoral immune diseases, in which pathogenic plasmablasts or long-lived plasma cells may sustain autoantibody or alloantibody production. Structurally, CD38 is classically described as a 46-kDa type II transmembrane glycoprotein with a short intracellular tail and a long extracellular domain containing enzymatic and antibody-binding sites (14, 15). This extracellular accessibility provides a structural basis for monoclonal antibody targeting, as illustrated by the crystal structure of CD38 in complex with daratumumab (15). Beyond plasma-cell biology, CD38 also contributes to immune-cell activation, inflammation, cell adhesion, migration, and homing, supporting its broader biological significance in immune-mediated conditions (16). The plasma-cell antigen landscape and the major mechanisms of action of daratumumab and isatuximab are summarized in **Figure 2**.

The enzymatic function of CD38 is central to its cross-disease relevance. Although CD38 was initially characterized as an ADP-ribosyl cyclase, it is now recognized as a major regulator of nicotinamide adenine dinucleotide (NAD+) and nicotinamide mononucleotide metabolism (14, 17). Through NAD+ consumption and the generation of calcium-mobilizing metabolites, CD38 can influence metabolic fitness, inflammatory signaling, and immune microenvironmental states (14, 16). In addition, CD38 membrane topology may be more complex than the classical type II configuration, with type III orientation also described in selected contexts. These features help explain why CD38-targeted therapy may have effects beyond direct depletion of CD38-high malignant plasma cells.

The antitumor activity of anti-CD38 monoclonal antibodies is mediated mainly through immune-effector mechanisms. These include antibody-dependent cellular cytotoxicity, antibody-dependent cellular phagocytosis, and complement-dependent cytotoxicity (18–20). Antibody-dependent cellular cytotoxicity depends on Fcγ receptor engagement on effector cells, particularly natural killer cells, whereas antibody-dependent cellular phagocytosis involves monocytes and macrophages that engulf antibody-coated target cells (19). Complement activation can further contribute to target-cell elimination, although the relative contribution of each pathway may differ among antibodies, disease settings, and effector-cell environments (18, 20).

Anti-CD38 therapy also has immunomodulatory effects. Daratumumab has been shown to reduce CD38-positive regulatory T cells, regulatory B cells, and myeloid-derived suppressor cell-like populations, while promoting effector T-cell expansion and increased T-cell receptor clonality (21). This immune remodeling is important because it links CD38-targeted therapy to more than cytotoxic plasma-cell depletion. In autoantibody-driven diseases and transplant-related alloimmunity, the therapeutic rationale may involve both depletion of pathogenic antibody-producing cells and modulation of immune-regulatory compartments.

Direct Fc-independent effects differ among anti-CD38 antibodies. Daratumumab is primarily characterized by immune-effector mechanisms, whereas isatuximab can induce direct cytotoxicity against myeloma cells *in vitro* through caspase-dependent apoptosis and lysosome-mediated non-apoptotic cell death (22). Recent data further suggest that isatuximab-induced direct cell death may involve FOXM1 downregulation, reactive oxygen species generation, and inactivation of NEK2 and UBE2T in 1q-positive myeloma cells (23).

These mechanisms help explain the established activity of anti-CD38 antibodies in plasma cell disorders. Outside multiple myeloma and light-chain amyloidosis, however, CD38 expression alone is insufficient to predict clinical activity because target density, effector-cell function, and disease biology may differ substantially across indications.

## 3 Approved and emerging anti-CD38 antibodies: mechanistic differences and clinical implications

From a pharmacologic perspective, the most clinically advanced CD38-targeting strategies are IgG1 cell-depleting monoclonal antibodies. Their main differences lie in epitope recognition, effects on CD38 enzymatic activity, Fc-mediated effector functions, route of administration, and the capacity to induce direct tumor-cell death.

Daratumumab was the first anti-CD38 monoclonal antibody to broadly reshape the treatment landscape of multiple myeloma. Its activity reflects the integration of complement-dependent cytotoxicity, antibody-dependent cellular cytotoxicity, antibody-dependent cellular phagocytosis, and immunomodulatory effects that include depletion of regulatory immune subsets and expansion of effector T cells (3, 19, 21, 24). Because of this broad mechanism profile, daratumumab has become the most widely validated anti-CD38 platform in plasma cell disorders.

Isatuximab is the second anti-CD38 antibody approved by the European Medicines Agency and the US Food and Drug Administration for multiple myeloma (3). It binds a distinct epitope on CD38 and more effectively inhibits the hydrolase and cyclase activities of CD38 through allosteric antagonism (25). In addition to Fc-dependent effector mechanisms, isatuximab can induce direct tumor-cell killing, including homotypic aggregation-associated apoptosis, and can promote natural killer cell- and T cell-mediated antitumor immune responses (20, 26). These features support its role as a clinically differentiated but mechanistically related alternative within the anti-CD38 class.

An important recent development is subcutaneous isatuximab. In the randomized phase III IRAKLIA study, 531 patients with relapsed or refractory multiple myeloma received fixed-dose subcutaneous isatuximab (1,400 mg) through an on-body injector or intravenous isatuximab (10 mg/kg), both in combination with pomalidomide and dexamethasone. Subcutaneous administration was non-inferior for overall response rate (71.1% vs 70.5%) and pharmacokinetic exposure, while infusion-related reactions were substantially less frequent than with intravenous administration (1.5% vs 25.0%) (27). These findings parallel the development of subcutaneous daratumumab, which similarly maintained efficacy and pharmacokinetic exposure while reducing administration-related reactions and treatment time compared with intravenous administration (28). The differing delivery approaches also illustrate the increasing importance of administration convenience within the anti-CD38 class.

Beyond daratumumab and isatuximab, newer anti-CD38 antibodies have entered disease-specific development outside conventional myeloma settings. Felzartamab (MOR202) has been prospectively evaluated in IgA nephropathy, primary membranous nephropathy, and kidney-transplant antibody-mediated rejection (29–31). Mezagitamab (TAK-079) has been studied in relapsed/refractory multiple myeloma and in a randomized phase 2 trial in primary immune thrombocytopenia (32–34). CM313 has entered clinical development in autoimmune disease, including randomized studies in primary immune thrombocytopenia and systemic lupus erythematosus (35, 36). These agents differ in epitope recognition, effects on CD38 enzymatic activity, Fc-dependent effector functions, direct cell-death activity, and clinical development; these characteristics are summarized in **Table 1**.

**Table 1:** Mechanistic and clinical characteristics of selected anti-CD38 monoclonal antibodies.

Antibody	Molecular and epitope features	Effect on CD38 enzymatic activity	Fc-dependent effector mechanisms	Direct cell death	Clinical development/status	Refs.
Daratumumab	Fully human IgG1κ anti-CD38 mAb; binds a discontinuous CD38 epitope that does not overlap with that of isatuximab.	Inhibits ADPR cyclase activity but not NAD+ hydrolase activity in CD38-expressing MM cells.	ADCC, CDC, and ADCP.	Induces programmed cell death after FcγR-mediated cross-linking.	Established treatment in MM, with extensive phase 3 evidence in relapsed/refractory and newly diagnosed disease.	(5, 15, 19, 24, 37–39)
Isatuximab	Humanized anti-CD38 mAb; binds a discontinuous epitope distinct from the daratumumab epitope.	Strongly inhibits CD38 ADP-ribosyl cyclase activity; structural analysis supports an allosteric mechanism.	ADCC, ADCP, and CDC; ADCP and CDC are more evident at higher CD38 expression.	Cross-linking-independent apoptosis has been observed in CD38-high preclinical models; direct apoptosis was not detected in MM cells with CD38 levels more typical of patient samples.	Established treatment in MM, with phase 3 evidence in relapsed/refractory and newly diagnosed disease.	(25, 26, 40, 41)
Felzartamab (MOR202)	Fully human IgG1λ anti-CD38 mAb.	Not characterized.	ADCC and ADCP; CDC was not observed in the reported studies.	Not characterized.	Investigational; studied in phase 1–2a R/R MM, phase 1b/2a PMN, randomized phase 2a IgAN, and randomized phase 2 kidney-transplant AMR.	(29–31, 42)
Mezagitamab (TAK-079)	Fully human IgG1λ anti-CD38 mAb; detailed epitope mapping has not been reported.	Not characterized.	ADCC, ADCP, and CDC have been demonstrated using a sequence-based TAK-079 analog.	Cross-linking-dependent cell death was observed with a TAK-079 analog in Daudi cells; clear activity was not seen without cross-linking.	Investigational; phase 1b data are available in R/R MM and randomized phase 2 data in ITP. MM development was not continued after phase 1b.	(33, 34, 43, 44)
CM313	IgG1κ anti-CD38 mAb; mutational studies indicate an epitope distinct from that of daratumumab.	Partially inhibits CD38 enzymatic activity in preclinical assays.	ADCC, CDC, and ADCP.	Cross-linking-dependent apoptosis has been reported in preclinical models.	Investigational; randomized phase 2 data in ITP and phase Ib/IIa data in SLE have been published.	(35, 36, 45)

Mechanistic findings are assay- and model-dependent. “Not characterized” indicates that the feature was not directly assessed in the cited studies. Findings based on antibody analogs are indicated where applicable. ADCC, antibody-dependent cellular cytotoxicity; ADCP, antibody-dependent cellular phagocytosis; ADPR, adenosine diphosphate ribose; AMR, antibody-mediated rejection; CDC, complement-dependent cytotoxicity; IgAN, immunoglobulin A nephropathy; ITP, immune thrombocytopenia; MM, multiple myeloma; PMN, primary membranous nephropathy; R/R, relapsed/refractory; SLE, systemic lupus erythematosus.

## 4 Clinical positioning of anti-CD38 immunotherapy in multiple myeloma and plasma cell disorders

The clinical development of anti-CD38 monoclonal antibodies in plasma cell disorders has followed a stepwise trajectory, moving from relapsed/refractory multiple myeloma to frontline treatment, maintenance intensification, and minimal residual disease-oriented strategies across the disease course (4, 6, 37). In AL amyloidosis, daratumumab-based therapy has further extended this platform into a distinct plasma cell-driven disorder in which organ outcomes are integral to treatment evaluation (7, 8). The pivotal and representative evidence supporting anti-CD38 therapy in plasma cell disorders is summarized in **Table 2**.

**Table 2:** Pivotal and representative clinical evidence for anti-CD38 monoclonal antibodies in multiple myeloma and AL amyloidosis.

Disease setting	Study/design (N)	Anti-CD38 regimen/comparator	Follow-up and key numerical efficacy	Major safety findings and key limitations	Refs.
RRMM	POLLUX; randomized, open-label phase 3; N = 569	D-Rd vs Rd	Median follow-up 79.7 mo; median OS 67.6 vs 51.8 mo; HR 0.73 (95% CI 0.58–0.91)	Grade 3/4 neutropenia 57.6% vs 41.6% and pneumonia 17.3% vs 11.0%. Protocol amendment allowed daratumumab after progression in the control arm; the trial also largely predates routine frontline anti-CD38 exposure.	(37)
RRMM	CASTOR; randomized, open-label phase 3; N = 498	D-Vd vs Vd	Median follow-up 72.6 mo; median OS 49.6 vs 38.5 mo; HR 0.74 (95% CI 0.59–0.92)	Grade 3/4 thrombocytopenia 46.1% vs 32.9%, neutropenia 13.6% vs 4.6%, and pneumonia 10.7% vs 10.1%. Control-arm patients could receive daratumumab after progression; contemporary applicability after prior frontline anti-CD38 exposure is uncertain.	(46)
RRMM	ICARIA-MM; randomized, open-label phase 3; N = 307	Isa-Pd vs Pd	Final OS follow-up 52.4 mo; median OS 24.6 vs 17.7 mo; HR 0.78 (95% CI 0.59–1.02)	In the final safety analysis, grade ≥3 neutropenia was 51% vs 35% and pneumonia 23% vs 21%. The final OS analysis did not meet the prespecified statistical significance criterion; subsequent daratumumab use was more frequent in the control arm.	(40, 47)
RRMM	IKEMA; randomized, open-label phase 3; N = 302	Isa-Kd vs Kd	Updated median PFS 35.7 vs 19.2 mo; HR 0.58 (95.4% CI 0.42–0.79). At final OS follow-up of 56.6 mo, median OS was NR vs 50.6 mo; HR 0.855 (95% CI 0.608–1.202)	Grade ≥3 pneumonia 18.6% vs 12.3% in the updated safety analysis; no new safety signal was identified at final OS follow-up. A statistically significant OS difference was not detected; the OS comparison was non-inferential because of hierarchical testing.	(48–50)
TI-NDMM	MAIA; randomized, open-label phase 3; N = 737	D-Rd vs Rd	Median follow-up 64.5 mo; median PFS 61.9 vs 34.4 mo, HR 0.55 (95% CI 0.45–0.67); median OS NR vs 65.5 mo, HR 0.66 (95% CI 0.53–0.83); MRD negativity 32.1% vs 11.1%	Grade 3/4 neutropenia 54.1% vs 37.0% and infections 42.6% vs 29.6%. Continuous-treatment design and enrollment before widespread use of frontline anti-CD38 quadruplets should be considered when extrapolating sequencing.	(4, 51)
TI-NDMM	ALCYONE; randomized, open-label phase 3; N = 706	D-VMP vs VMP	Median follow-up 86.7 mo; median OS 83.0 vs 53.6 mo; HR 0.65 (95% CI 0.53–0.80)	Grade 3/4 neutropenia 40% vs 39% and thrombocytopenia 35% vs 38%; serious treatment-related AEs 21% vs 16%. VMP is less representative of contemporary frontline practice in many regions.	(52, 53)
TI-NDMM	IMROZ; randomized, open-label phase 3; N = 446	Isa-VRd → Isa-Rd vs VRd → Rd	Median follow-up 59.7 mo; 60-mo PFS 63.2% vs 45.2%; HR 0.60 (98.5% CI 0.41–0.88); ≥CR 74.7% vs 64.1%; MRD-negative CR 55.5% vs 40.9%	No new safety signals; serious AEs and AEs leading to discontinuation were reported as similar between groups. Patients >80 years were excluded, and OS remained immature at the reported analysis.	(54, 55)
Frail NDMM	IFM2017-03; randomized, open-label phase 3; N = 295	Daratumumab + lenalidomide with dexamethasone limited to cycles 1–2 vs Rd	Median follow-up 46.3 mo; median PFS 53.4 vs 22.5 mo; HR 0.51 (95% CI 0.37–0.70)	Grade 3–5 neutropenia 55% vs 24% and infections 19% vs 21%; serious AEs 63% vs 69%. Deliberately selected frail population (median age 81 y) limits generalization to fitter patients.	(56)
TE-NDMM	CASSIOPEIA; two-stage randomized, open-label phase 3; N = 1,085 at first randomization; n=886 rerandomized for maintenance	D-VTd vs VTd; then daratumumab maintenance vs observation	Median follow-up 80.1 mo from first and 70.6 mo from second randomization; from second randomization, median PFS was NR vs 45.8 mo; HR 0.49 (95% CI 0.40–0.59)	In the maintenance safety analysis, grade 3/4 lymphopenia was 4% vs 2% and neutropenia 2% vs 2%. Important limitations are the thalidomide-based induction backbone, observation rather than lenalidomide as the maintenance comparator, and the two-stage randomization.	(6, 57)
TE-NDMM	PERSEUS; randomized, open-label phase 3; N = 709	D-VRd → D-R maintenance vs VRd → R maintenance	Median follow-up 47.5 mo; 48-mo PFS 84.3% vs 67.7%; HR 0.42 (95% CI 0.30–0.59); ≥CR 87.9% vs 70.1%; MRD negativity 75.2% vs 47.5%	Grade 3/4 neutropenia 62.1% vs 51.0% and thrombocytopenia 29.1% vs 17.3%. Daratumumab was incorporated across induction, consolidation, and maintenance, so the contribution of individual treatment phases cannot be isolated; OS remains immature.	(5)
TE-NDMM	GMMG-HD7; randomized, open-label phase 3; N = 662 randomized; n=660 in the ITT analysis	Isa-RVd vs RVd induction, followed by ASCT and second maintenance randomization	Median follow-up 47 mo; PFS HR 0.70 (95% CI 0.52–0.95); 3-y PFS 83% vs 75%; post-transplant MRD negativity 66% vs 48%	During induction, grade 3/4 neutropenia 23% vs 7% and infections 12% vs 10%. Subsequent maintenance rerandomization complicates attribution of long-term PFS solely to induction; benefit in high-risk cytogenetic subgroups remains uncertain.	(41, 58)
Post-ASCT maintenance	AURIGA; randomized phase 3; N = 200	D-R vs R maintenance in MRD-positive, anti-CD38–naïve patients	Median follow-up 32.3 mo; 12-mo MRD-negative conversion 50.5% vs 18.8%; PFS HR 0.53 (95% CI 0.29–0.97); estimated 30-mo PFS 82.7% vs 66.4%	Grade 3/4 cytopenias 54.2% vs 46.9% and infections 18.8% vs 13.3%. Restricted to MRD-positive, anti-CD38–naïve patients; PFS was an interim analysis and did not cross the prespecified stopping boundary, and applicability after frontline anti-CD38 therapy remains uncertain.	(59)
MRD-directed post-frontline therapy	DART4MM; prospective, open-label, single-arm phase 2; N = 50 treated	Daratumumab monotherapy; MRD-negative patients stopped at 6 mo, MRD-positive patients continued up to 24 mo	Median follow-up 50 mo; MRD negativity was 30% at 6 mo and 22% at 24 mo; overall median PFS was 45 mo. In an exploratory within-cohort analysis, median PFS was 61 vs 26 mo for patients ever vs never achieving MRD negativity (HR 0.24, 95% CI 0.10–0.56)	Grade 3/4 lymphopenia 24%; grade 3/4 infections 2%. Small, selected, single-arm study; the association between MRD conversion and PFS does not establish the efficacy of MRD-guided treatment adaptation.	(60)
Newly diagnosed AL amyloidosis	ANDROMEDA; randomized, open-label phase 3; N = 388	D-VCd → daratumumab vs VCd	Final median follow-up 61.4 mo; hematologic CR 59.5% vs 19.2%; MOD-PFS HR 0.44 (95% CI 0.31–0.63); OS HR 0.62 (95% CI 0.42–0.90)	In the initial randomized safety analysis, grade 3/4 lymphopenia was 13.0% vs 10.1% and pneumonia 7.8% vs 4.3%. Mayo stage IIIb disease was excluded, limiting generalization to the highest-risk cardiac population.	(7, 8, 61, 62)
Mayo stage IIIb AL amyloidosis	Multicenter retrospective study; N = 119 consecutive patients; 98 included in upfront-treatment comparison	Upfront daratumumab-containing vs bortezomib-based vs other regimens	At a median follow-up of 31.3 mo, 6-mo OS was 70% vs 51% vs 25%; 6-mo hematologic ORR was 66.7% vs 40.7% for daratumumab- vs bortezomib-based therapy	Therapy-associated adverse events were not systematically recorded. Retrospective design, missing data, selection/referral bias, and sequential addition of treatment components limit causal interpretation.	(63)
Mayo stage IIIb AL amyloidosis	EMN22; prospective, single-arm phase 2; N = 40	Daratumumab monotherapy; bortezomib+dexamethasone permitted after inadequate response	Primary endpoint assessed at 6 mo; 6-mo OS 65.0% (95% CI 48.2–77.6); median OS 10.4 mo; hematologic response ≥PR by 6 mo 75.0%; cardiac response at 6 mo 30.0%	Common serious AEs included cardiac failure 25.0%, sudden cardiac death 10.0%, and acute kidney injury 7.5%. Single-arm design and addition of bortezomib+dexamethasone in 25% of patients limit attribution to daratumumab monotherapy.	(64)
Relapsed/refractory AL amyloidosis	SWOG S1702; multicenter, single-arm phase 2; N = 43 registered, n=35 response-evaluable	Isatuximab monotherapy	Median follow-up 21.7 mo; hematologic ORR 77.1%; ≥VGPR 57%; renal response 50% and cardiac response 57% among evaluable patients	Treatment-related grade ≥3 lymphopenia 8.5% and infection 6%. Small single-arm study without comparator; only 35 patients were response-evaluable.	(65)

This table summarizes pivotal and representative studies discussed in the text and is not exhaustive. Where multiple analyses of the same study were available, the most mature published efficacy data were prioritized. Safety findings are reported as published and should not be compared directly across trials; in combination studies, adverse events reflect the full treatment regimen and should not be attributed to the anti-CD38 component alone. Outcomes from single-arm and retrospective studies should be interpreted in light of their nonrandomized design, and inclusion does not imply regulatory approval or guideline endorsement of a specific regimen or treatment-adaptation strategy. AE, adverse event; AL, immunoglobulin light-chain amyloidosis; ASCT, autologous stem-cell transplantation; CI, confidence interval; CR, complete response; PR, partial response; R, lenalidomide; D-R, daratumumab plus lenalidomide; D-Rd, daratumumab plus lenalidomide and dexamethasone; D-VCd, daratumumab plus bortezomib, cyclophosphamide, and dexamethasone; D-Vd, daratumumab plus bortezomib and dexamethasone; D-VMP, daratumumab plus bortezomib, melphalan, and prednisone; D-VRd, daratumumab plus bortezomib, lenalidomide, and dexamethasone; D-VTd, daratumumab plus bortezomib, thalidomide, and dexamethasone; HR, hazard ratio; Isa-Kd, isatuximab plus carfilzomib and dexamethasone; Isa-Pd, isatuximab plus pomalidomide and dexamethasone; Isa-Rd, isatuximab plus lenalidomide and dexamethasone; Isa-RVd/Isa-VRd, isatuximab plus lenalidomide, bortezomib, and dexamethasone; ITT, intention-to-treat; Kd, carfilzomib plus dexamethasone; MM, multiple myeloma; MOD-PFS, major organ deterioration progression-free survival; MRD, minimal residual disease; NDMM, newly diagnosed multiple myeloma; NR, not reached; ORR, overall response rate; OS, overall survival; Pd, pomalidomide plus dexamethasone; PFS, progression-free survival; Rd, lenalidomide plus dexamethasone; RRMM, relapsed/refractory multiple myeloma; TE-NDMM, transplant-eligible newly diagnosed multiple myeloma; TI-NDMM, transplant-ineligible newly diagnosed multiple myeloma; VCd, bortezomib, cyclophosphamide, and dexamethasone; Vd, bortezomib plus dexamethasone; VGPR, very good partial response; VMP, bortezomib, melphalan, and prednisone; VRd/RVd, bortezomib, lenalidomide, and dexamethasone; VTd, bortezomib, thalidomide, and dexamethasone.

### 4.1 Multiple myeloma

Anti-CD38 monoclonal antibodies were first clinically validated in relapsed/refractory multiple myeloma (RRMM), where they established the principle that targeting CD38 could deepen responses and improve long-term outcomes when added to standard backbone regimens. The POLLUX and CASTOR studies demonstrated the value of daratumumab in both immunomodulatory drug- and proteasome inhibitor-based relapse regimens, whereas ICARIA-MM and IKEMA extended this principle to isatuximab-containing combinations (**Table 2**) (37, 40, 46–49). Collectively, these studies showed that CD38-targeted therapy provides clinically meaningful benefit across different relapse backbones in anti-CD38-naive RRMM. However, these trials were largely conducted before anti-CD38 antibodies became widely incorporated into frontline therapy. Thus, in contemporary practice, the key question has shifted from whether anti-CD38 therapy is active in RRMM to how it should be sequenced, reintroduced, or combined after prior CD38-targeted exposure.

In transplant-ineligible newly diagnosed multiple myeloma (TI-NDMM), anti-CD38 therapy has had particular clinical impact because older or frail patients often have fewer opportunities for effective salvage therapy. The MAIA study established daratumumab, lenalidomide, and dexamethasone (D-Rd) as a highly effective continuous frontline approach, with improved progression-free survival (PFS), overall survival (OS), and minimal residual disease (MRD) outcomes compared with lenalidomide and dexamethasone alone (4, 51). ALCYONE showed that daratumumab also improved outcomes when added to bortezomib, melphalan, and prednisone, indicating that the value of CD38 targeting is not restricted to an immunomodulatory drug-based regimen (52, 53). More recent studies have moved the field toward both intensification and de-intensification strategies. IMROZ supports isatuximab-containing quadruplet therapy in selected TI-NDMM patients, whereas IFM2017–03 suggests that, in frail patients, daratumumab-based treatment may be optimized by limiting dexamethasone exposure while preserving efficacy (54, 56). These data indicate that anti-CD38 therapy in TI-NDMM should not be viewed as a single uniform approach, but rather as a platform that can be adapted according to frailty, comorbidities, treatment goals, and tolerability.

In transplant-eligible newly diagnosed multiple myeloma (TE-NDMM), the incorporation of anti-CD38 monoclonal antibodies has changed both the composition and the goals of frontline therapy. The CASSIOPEIA study first demonstrated that adding daratumumab to bortezomib, thalidomide, and dexamethasone improved response depth and PFS, and that daratumumab maintenance could further improve disease control (6). Although CASSIOPEIA used a thalidomide-based induction regimen and observation rather than lenalidomide as the maintenance comparator, it established the feasibility of integrating anti-CD38 therapy across induction, consolidation, and maintenance. PERSEUS then embedded daratumumab into the more contemporary bortezomib, lenalidomide, and dexamethasone backbone, showing improved PFS and higher rates of complete response, MRD negativity, and sustained MRD negativity (5). The GMMG-HD7 study further extended quadruplet induction evidence from daratumumab to isatuximab, whereas AURIGA supported daratumumab plus lenalidomide as a maintenance intensification strategy for MRD-positive patients after autologous stem-cell transplantation (ASCT) (41, 59). More recently, the phase 2 DART4MM study evaluated an MRD-directed daratumumab strategy in daratumumab-naïve patients who had achieved at least a very good partial response but remained MRD-positive after first-line therapy. Among 50 treated patients, 30% converted to MRD negativity after 6 months of daratumumab, and 22% remained MRD negative at 24 months. Patients who achieved MRD negativity at least once had longer median PFS than those who remained MRD positive (61 vs 26 months) (60). The phase 3 MIDAS trial also used post-induction MRD status to define randomized consolidation strategies after Isa-KRd induction; however, it evaluated a multi-component consolidation strategy rather than anti-CD38 treatment duration alone, and follow-up remains relatively short (66). These studies support MRD-based treatment adaptation as an area of prospective investigation, but do not establish routine MRD-guided escalation or discontinuation of anti-CD38 therapy.

Taken together, anti-CD38 monoclonal antibodies have evolved from relapse-directed therapy to frontline quadruplet and maintenance-intensification strategies, while MRD-guided treatment adaptation remains investigational. Future challenges include sequencing after frontline CD38 exposure, retreatment, de-escalation, and integration with emerging immune-based approaches.

### 4.2 AL amyloidosis

Immunoglobulin light-chain (AL) amyloidosis is also driven by clonal plasma cells, but its clinical risk differs fundamentally from that of multiple myeloma. Disease morbidity and early mortality are determined largely by organ damage caused by misfolded light-chain deposition, particularly in the heart and kidneys. Therefore, the therapeutic objective is not simply to reduce plasma-cell burden, but to suppress pathogenic light-chain production rapidly enough to permit organ stabilization or recovery (7).

The pivotal ANDROMEDA study established daratumumab, bortezomib, cyclophosphamide, and dexamethasone (D-VCd) as a frontline anti-CD38-based regimen for newly diagnosed AL amyloidosis. Compared with bortezomib, cyclophosphamide, and dexamethasone (VCd) alone, D-VCd improved hematologic and organ response rates and prolonged time to major organ deterioration, hematologic progression, or death (7, 61). Final analyses further supported the durability and clinical relevance of this benefit, including improvements in major organ deterioration progression-free survival (MOD-PFS) and overall survival (OS) (8). Thus, in AL amyloidosis, anti-CD38 therapy has moved beyond extrapolation from myeloma and has become part of a disease-specific therapeutic paradigm centered on rapid clonal suppression and organ outcomes.

Several issues remain clinically important. First, ANDROMEDA excluded patients with Mayo stage IIIb disease, a group with particularly high early mortality. Retrospective data suggest that frontline daratumumab-containing therapy may improve responses and survival in this high-risk population, but these findings require cautious interpretation because they are not derived from randomized prospective evidence (63). Second, as daratumumab-based therapy becomes a frontline backbone, risk stratification in AL amyloidosis is evolving beyond organ staging alone. Cytogenetic features may influence outcomes under modern therapy; for example, 1q gain or amplification was associated with inferior hematologic event-free survival in a daratumumab-based frontline setting, whereas t(11;14) did not show an adverse prognostic impact in that study (67). These observations support a more integrated approach combining organ risk and clonal biology.

Isatuximab has also been evaluated in relapsed or refractory AL amyloidosis, but the evidence base remains much less mature than that for daratumumab (65). At present, the strongest clinical role for anti-CD38 therapy in AL amyloidosis remains frontline daratumumab-based treatment. Future studies should define optimal strategies for patients with very advanced cardiac involvement, cytogenetically high-risk disease, relapsed or refractory disease after prior daratumumab exposure, and treatment approaches that balance rapid clonal control with organ-specific tolerability.

### 4.3 Other plasma cell-related disorders: limited evidence, but clear mechanistic rationale

Compared with multiple myeloma and immunoglobulin light-chain amyloidosis, the evidence supporting anti-CD38 therapy in other plasma cell-related disorders remains limited. In primary plasma cell leukemia, anti-CD38-based regimens have a clear mechanistic rationale and preliminary clinical activity, but available data are mainly retrospective or derived from small clinical experiences (68). Similarly, early positive signals have been reported in rare plasma cell disorders such as POEMS syndrome, but evidence remains insufficient to define a standard clinical role (69). These observations suggest that CD38 targeting may be biologically plausible across a broader spectrum of plasma cell disorders, although current support is not comparable to the mature randomized evidence available in multiple myeloma and light-chain amyloidosis.

Overall, anti-CD38 monoclonal antibodies have become a foundational therapeutic platform in plasma cell disorders, with the strongest evidence in multiple myeloma and light-chain amyloidosis. In rarer plasma cell-related diseases, their role remains biologically plausible but requires disease-specific validation.

## 5 Beyond established plasma cell disorders: emerging hematologic and humoral immune applications

Beyond established plasma cell disorders, anti-CD38 therapy has been explored in non-plasma cell hematologic malignancies and a range of immune-mediated diseases. However, the evidence varies substantially across indications, from prospective clinical trials to retrospective studies and case-based reports. These emerging applications are therefore discussed according to biological rationale, strength of clinical evidence, major limitations, and current clinical relevance (**Table 3**).

**Table 3:** Evidence maturity and current clinical status of emerging anti-CD38 applications beyond plasma cell disorders.

Disease area	Representative indications	Biological rationale	Evidence base described in this review	Current clinical/regulatory status*	Refs.
Non-plasma cell acute leukemias	R/R T-ALL/T-LBL, including ETP-ALL; AML	CD38 expression provides a rationale for anti-CD38 targeting in T-ALL/T-LBL. In AML, IFN-γ can induce CD38 expression in leukemia-stem-cell–enriched blasts, supporting an inducible-target rationale.	Prospective evidence includes the phase 2 DELPHINUS study and a 21-patient single-arm phase 1 study of daratumumab plus venetoclax and CAGE, both reporting clinical activity in R/R T-ALL/T-LBL; the ETP-ALL signal derives from a small subgroup. AML evidence includes mechanistic/preclinical work and small nonrandomized clinical studies of daratumumab-containing salvage or HSCT-preparative regimens.	T-ALL/T-LBL: prospective investigational, with daratumumab use off-label. ETP-ALL: small-subgroup clinical signal. AML: experimental; clinical evidence remains limited and nonrandomized.	(9, 70–76)
Selected lymphomas	PBL; selected CD20-negative/CD38-positive DLBCL	CD38 expression in plasmablastic LBCL/PBL and CD20-negative/CD38-positive DLBCL provides a target rationale for daratumumab-containing therapy.	In a phase 2 study of daratumumab monotherapy in R/R DLBCL, FL, and MCL selected for ≥50% CD38 expression, the DLBCL and FL cohorts showed low response rates and met prespecified futility criteria; small series subsequently reported responses in plasmablastic LBCL/PBL and CD20-negative/CD38-positive DLBCL.	Off-label/experimental; selected-phenotype responses remain hypothesis-generating and do not establish a standard role.	(77–80)
Autoimmune cytopenias	Persistent/chronic ITP; refractory AIHA	Long-lived plasma cells can sustain pathogenic antibody production, providing a rationale for CD38-directed depletion.	ITP now has randomized phase 2 evidence for CM313 and mezagitamab and open-label phase 2 evidence for daratumumab. In AIHA, daratumumab evidence remains predominantly retrospective.	ITP: prospective investigational development; daratumumab use remains off-label. AIHA: retrospective/off-label evidence. Routine anti-CD38 therapy is not established.	(34, 35, 81–87)
Systemic autoimmune diseases	SLE, including refractory lupus nephritis; primary Sjögren disease	Persistent antibody-secreting cells and long-lived plasma cells provide a rationale for CD38-directed depletion.	SLE evidence includes a randomized phase Ib/IIa CM313 study and a single-arm phase 2 daratumumab study. Evidence in refractory lupus nephritis and primary Sjögren disease remains limited to small case series or case-based reports.	SLE: prospective investigational development; daratumumab use remains off-label. Lupus nephritis and Sjögren disease: off-label/experimental.	(36, 88–94)
Immune-mediated kidney diseases	IgAN; anti-PLA2R-positive PMN	Pathogenic immunoglobulin or autoantibody production provides a rationale for targeting antibody-secreting cells.	Felzartamab has been evaluated in a randomized phase 2a study in IgAN and an open-label phase 1b/2a study in anti-PLA2R-positive PMN, with proteinuria and immunologic response signals reported.	Prospective investigational development; routine use is not established. Felzartamab has FDA orphan designation for membranous nephropathy but is not FDA-approved for that indication.	(30, 31)
Solid-organ transplant alloimmunity	Late kidney-transplant AMR; kidney or heart-transplant desensitization	Donor-specific antibody-producing plasma cells and CD38-expressing NK cells provide targets for CD38-directed intervention.	A randomized phase 2 trial of felzartamab in late kidney AMR showed improvement in morphologic and biomarker measures but also recurrence after treatment withdrawal. Daratumumab desensitization studies in kidney and heart transplantation remain small, prospective, and nonrandomized.	Kidney AMR: randomized phase 2 investigational evidence; felzartamab is not FDA-approved for this indication. Daratumumab-based desensitization remains off-label and investigational.	(11, 29, 95–101)
HSCT-related antibody-mediated complications	PRCA after ABO-mismatched allogeneic HCT; refractory post-HSCT immune cytopenias	Persistent recipient-derived antibody-producing cells may sustain pathogenic antibodies after transplantation.	An international EBMT observational study described outcomes in 45 patients treated with daratumumab for post-HCT PRCA; evidence for other post-HSCT immune cytopenias remains case-based.	Post-HCT PRCA: off-label; multicenter observational evidence is available. Other immune cytopenias: exploratory/case-based.	(86, 102–104)
Antibody-mediated neurologic diseases	Severe refractory autoimmune encephalitis; selected antibody-associated neuropathies, including CIDP	Treatment-resistant long-lived plasma cells provide a rationale for CD38-directed depletion.	A retrospective single-center series included seven severely pretreated patients with heterogeneous antibody-mediated neurologic diseases; grade ≥3 treatment-related toxicities occurred in five patients, including one death. Additional CIDP evidence remains at case-report level.	Off-label/experimental; evidence remains at case-series or case-report level, with no established routine clinical role.	(105–107)

This table summarizes representative emerging anti-CD38 applications beyond established plasma cell disorders and distinguishes biological rationale, evidence maturity, and current clinical status. “Prospective investigational” denotes evidence from prospective clinical trials without established routine use; “observational” denotes nonrandomized cohort evidence; “off-label” denotes use of an approved agent outside its labeled indication; and “experimental” denotes applications supported mainly by preclinical, small nonrandomized clinical, case-series, or case-report evidence. Evidence classifications reflect the design and maturity of the cited primary studies and do not constitute treatment recommendations. *Regulatory statements, including “off-label” and “not FDA-approved,” refer to U.S. FDA status as of August 1, 2026; regulatory status may differ by jurisdiction. AIHA, autoimmune hemolytic anemia; AML, acute myeloid leukemia; AMR, antibody-mediated rejection; CIDP, chronic inflammatory demyelinating polyradiculoneuropathy; DLBCL, diffuse large B-cell lymphoma; EBMT, European Society for Blood and Marrow Transplantation; ETP-ALL, early T-cell precursor acute lymphoblastic leukemia; HCT, hematopoietic cell transplantation; HSCT, hematopoietic stem cell transplantation; IgAN, immunoglobulin A nephropathy; ITP, immune thrombocytopenia; LBCL, large B-cell lymphoma; NK, natural killer; PBL, plasmablastic lymphoma; PMN, primary membranous nephropathy; PRCA, pure red cell aplasia; R/R, relapsed/refractory; SLE, systemic lupus erythematosus; T-ALL/T-LBL, T-cell acute lymphoblastic leukemia/lymphoblastic lymphoma.

### 5.1 Non-plasma cell hematologic malignancies

Compared with multiple myeloma and immunoglobulin light-chain amyloidosis, the development of anti-CD38 therapy in non-plasma cell hematologic malignancies remains at an earlier and more exploratory stage. The main limitation is that CD38 expression is more heterogeneous and less lineage-defining than in plasma cell disorders. Therefore, clinical activity cannot be inferred from CD38 expression alone; it must be interpreted in relation to target density, disease biology, timing of therapy, and the availability of competent immune effector mechanisms.

In acute myeloid leukemia (AML), CD38 can be detected on leukemic blasts, but the leukemic stem cell-enriched compartment is often characterized by a CD34-positive/CD38-negative phenotype, limiting the rationale for passive depletion based solely on baseline CD38 expression (70). Mechanistic studies suggest that interferon-gamma can induce CD38 expression in AML blasts and leukemia stem cell-enriched populations, supporting the concept of CD38 as an inducible immunotherapeutic target rather than a stable lineage-defining antigen in AML (71). Clinically, the evidence remains limited to case reports, small retrospective studies, early combination approaches, and transplantation-related strategies (72, 73). These findings support further investigation, but anti-CD38 therapy remains experimental in AML and has no established independent clinical role.

The strongest non-plasma cell hematologic malignancy signal is currently in T-cell acute lymphoblastic leukemia/lymphoblastic lymphoma (T-ALL/T-LBL), where CD38 expression is more directly relevant (9, 74). The DELPHINUS study showed that daratumumab combined with chemotherapy had an acceptable safety profile and antileukemic activity in pediatric and young adult patients with relapsed or refractory T-ALL/T-LBL, with some patients able to proceed to allogeneic transplantation (9). Importantly, response appears to be influenced by target biology: baseline CD38 expression may be associated with response, whereas CD38 expression can decrease during therapy (74). Early T-cell precursor acute lymphoblastic leukemia (ETP-ALL) is also a biologically compelling high-risk subgroup, but the current evidence remains largely case-based and requires prospective validation (75).

In lymphoma, the available evidence argues against unselected anti-CD38 use. Daratumumab monotherapy showed limited activity in relapsed or refractory mantle cell lymphoma, diffuse large B-cell lymphoma (DLBCL), and follicular lymphoma (77). More plausible settings are those with plasmablastic differentiation, low or absent CD20 expression, high CD38 expression, or selected refractory contexts in which CD38 provides a biologically plausible target. Early signals have been reported in plasmablastic lymphoma (PBL) and selected CD20-negative/CD38-positive relapsed or refractory DLBCL, including daratumumab-based combinations in selected CD38-positive or plasmablastic phenotypes (78, 79). However, these observations remain based on small studies or case reports and are insufficient to change standard lymphoma treatment algorithms.

### 5.2 Autoimmune cytopenias and immune-mediated hematologic disorders

Among non-malignant diseases, autoimmune cytopenias represent one of the most hematology-relevant areas for anti-CD38 development. The biological rationale is distinct from conventional B-cell depletion: in refractory disease, pathogenic antibodies may be sustained not only by circulating B cells, but also by plasmablasts and long-lived plasma cells that are not adequately eliminated by standard immunosuppressive or anti-CD20-based strategies. CD38-targeted therapy therefore offers a mechanistically different approach aimed at depleting antibody-producing cells and modifying the humoral immune process underlying persistent cytopenia.

Primary immune thrombocytopenia (ITP) is currently the most actively developing autoimmune cytopenia indication for anti-CD38 therapy. The rationale is supported by the persistence of autoreactive plasmablasts and long-lived plasma cells in some patients with refractory or relapsed disease (82). CM313, a novel anti-CD38 monoclonal antibody, induced rapid platelet responses in patients with relapsed or refractory ITP and subsequently showed improved treatment response compared with placebo in a randomized phase 2 trial (35, 83). Daratumumab has also demonstrated clinical activity in heavily pretreated ITP (81). These studies provide prospective clinical evidence for CD38 targeting in ITP, although routine clinical use is not established. Nevertheless, optimal patient selection, dosing schedule, retreatment strategy, and long-term effects on immunoglobulin levels and infection risk remain insufficiently defined.

Autoimmune hemolytic anemia (AIHA) provides a related but less mature example. AIHA is a heterogeneous disorder in which relapsed or refractory disease may, in selected cases, be maintained by long-lived plasma cells producing pathogenic autoantibodies (84). A multicenter retrospective study suggested that daratumumab may improve hemolytic anemia and cold-induced circulatory symptoms in refractory warm autoimmune hemolytic anemia and cold agglutinin disease, with a rapid onset of action in some patients (85). Anti-CD38 therapy has also been explored in antibody-mediated erythroid complications after allogeneic hematopoietic stem cell transplantation, including pure red cell aplasia and AIHA, where residual host plasma cells may contribute to persistent antibody production (86). However, compared with ITP, the evidence in AIHA remains mainly retrospective or case-based.

Overall, evidence is more mature in ITP than in AIHA. Anti-CD38 therapy remains investigational or off-label in both settings, and patient selection, treatment duration, and long-term infectious and immunologic safety require further study.

### 5.3 Transplant-related alloimmunity and antibody-mediated complications

Transplantation provides a biologically plausible setting for CD38 targeting because persistent plasma cells can sustain alloantibody-mediated complications despite B-cell-directed or conventional immunosuppressive approaches (11, 29). However, the strength of evidence differs substantially across transplant settings.

In HSCT, the most substantial evidence concerns pure red cell aplasia (PRCA) after ABO-incompatible transplantation. An international EBMT observational study reported clinically meaningful responses to daratumumab in 45 patients with post-transplant PRCA (102). Evidence for other post-HSCT immune cytopenias remains largely case-based (86, 103, 104).

In solid-organ transplantation, a randomized phase 2 study of felzartamab in late kidney antibody-mediated rejection reported improvements in morphologic and biomarker measures, although recurrence after treatment withdrawal indicates that the durability of benefit remains uncertain (29). Daratumumab- or isatuximab-based desensitization has also been explored in kidney and heart transplantation, but available studies remain small and nonrandomized (98–101).

Overall, anti-CD38 therapy remains investigational or off-label in transplantation. Current evidence is insufficient to define routine use, optimal treatment duration, or long-term infectious and immunologic safety.

### 5.4 Other humoral immune diseases

Beyond autoimmune cytopenias and transplantation, anti-CD38 therapy has been explored in systemic autoimmune diseases, immune-mediated kidney diseases, and antibody-mediated neurologic disorders. The evidence in these disorders remains heterogeneous and generally less mature than that in plasma cell disorders or autoimmune cytopenias.

In systemic autoimmune diseases, including systemic lupus erythematosus, lupus nephritis, antineutrophil cytoplasmic antibody-associated vasculitis, and Sjögren disease, early studies and case reports suggest that CD38-directed therapy may reduce antibody-secreting cells, autoantibody burden, and disease activity in selected refractory cases (36, 88–93). Immune-mediated kidney diseases provide a more disease-specific development path: phase 2 studies of felzartamab in immunoglobulin A nephropathy and primary membranous nephropathy have reported reductions in proteinuria and relevant immunoglobulin or autoantibody markers (30, 31). Antibody-mediated neurologic diseases remain earlier in development, with only preliminary reports in severe anti-N-methyl-D-aspartate receptor encephalitis and chronic inflammatory demyelinating polyradiculoneuropathy (105, 106).

Overall, these diseases illustrate the biological breadth of CD38 targeting, but they should be interpreted as proof-of-concept for humoral immune modulation rather than established clinical indications. Broader development will require prospective evidence, disease-specific biomarkers, and careful assessment of immunoglobulin depletion, infection risk, and cumulative immunosuppression.

## 6 Clinical implementation: safety, laboratory interference, and resistance

The clinical value of anti-CD38 monoclonal antibodies depends not only on efficacy, but also on safe implementation, correct laboratory interpretation, and an understanding of response heterogeneity. Because CD38 is expressed on normal antibody-secreting cells and other immune-cell subsets, anti-CD38 therapy can lower polyclonal immunoglobulin levels and alter immune-cell compartments (21, 81). In multiple myeloma, infection and cytopenia rates should be interpreted at the regimen level: shared-backbone randomized trials and meta-analyses show that adding an anti-CD38 antibody can increase some infectious and hematologic toxicities (47, 51, 108). Background risk is also influenced by disease-related immune dysfunction, prior therapy, corticosteroids, and partner drugs (109). Laboratory interference with pretransfusion testing and monoclonal-protein assessment is a distinct class-related effect (110–112). In addition, resistance may arise through reduced target availability, complement-inhibitory mechanisms, immune-effector dysfunction, or broader remodeling of the bone marrow immune microenvironment (113–115). These issues are summarized in **Table 4** and discussed below from a practical clinical perspective.

**Table 4:** Practical safety, laboratory, and resistance considerations for anti-CD38 monoclonal antibody therapy.

Issue	Mechanism or basis	Clinical consequence	Practical management or implication	Refs.
Administration-related reactions	Early reactions related to intravenous or subcutaneous administration, occurring mainly during the first administration	Infusion- or injection-related symptoms; lower early reaction rates have been reported with subcutaneous daratumumab	Premedication and monitoring during early dosing; use of subcutaneous formulation when appropriate	(28)
Hypogammaglobulinemia	Depletion of normal antibody-secreting cells and reduction of polyclonal immunoglobulins	Reduced immunoglobulin levels and potentially impaired humoral immunity	Monitor immunoglobulin levels; consider immunoglobulin replacement in selected patients with recurrent or severe infections	(36, 81, 108, 109, 116, 117)
Infection risk	Multifactorial: anti-CD38-related humoral immune effects, disease-related immune dysfunction, prior therapy, corticosteroids, and partner drugs	Increased risk of respiratory and severe infections, including pneumonia	Assess regimen- and patient-specific risk; vaccination, antiviral prophylaxis, and infection surveillance as appropriate	(108, 109, 116, 117)
Cytopenias	Multifactorial and regimen-dependent; myelosuppressive partner drugs, prior therapy, marrow reserve, and disease burden contribute	Neutropenia and thrombocytopenia; rates may increase after anti-CD38 addition in some shared-backbone trials	Interpret at the regimen level; monitor blood counts and use supportive care or partner-drug modification when appropriate rather than automatic anti-CD38 discontinuation	(47, 51, 109)
Pretransfusion testing interference	Binding of anti-CD38 antibodies to low-level CD38 on reagent red blood cells, causing positive indirect antiglobulin tests and panreactivity	Delayed or complicated antibody screening, antibody identification, and crossmatching	Baseline ABO/RhD typing and antibody screening before therapy; extended red cell phenotyping or genotyping when feasible; early communication with transfusion services	(110)
Mitigation of blood bank interference	Dithiothreitol treatment of reagent red blood cells can remove daratumumab-related panreactivity but destroys Kell antigens	Compatibility testing can be clarified, but Kell status must be considered	Use dithiothreitol-treated reagent red blood cells; provide Kell-negative red blood cells if Kell phenotype is unknown	(118)
Isatuximab-related testing interference	Isatuximab can also interfere with indirect antiglobulin testing, with patterns that may differ from daratumumab	Potential compatibility-testing complexity during transfusion support	Inform transfusion services of anti-CD38 exposure and apply appropriate mitigation strategies	(111)
Serum protein electrophoresis and immunofixation interference	Daratumumab is an IgG-kappa monoclonal antibody and may appear as a monoclonal band	False-positive M-protein signal or incorrect complete response assessment, especially in IgG-kappa myeloma	Interpret results in the context of treatment history; use daratumumab-specific immunofixation assays when needed	(112, 119)
Mass spectrometry-based response assessment	Therapeutic monoclonal antibodies can be distinguished from endogenous monoclonal proteins by molecular mass	More accurate response assessment during monoclonal antibody therapy	Consider mass spectrometry-based methods where available to resolve therapeutic antibody interference	(120, 121)
Target modulation and complement-inhibitory mechanisms	Reduced CD38 expression, lower baseline CD38 density, or increased complement-inhibitory proteins such as CD55 and CD59	Reduced sensitivity to anti-CD38 therapy and impaired complement-dependent cytotoxicity	Associated with response and resistance, but not validated for routine treatment selection or sequencing	(113, 122)
Genomic antigen escape	Biallelic disruption of the CD38 locus or altered CD38 variants in resistant disease	Stable resistance to anti-CD38 antibodies in a subset of patients	Potential resistance mechanism; not validated as a routine clinical biomarker	(114)
Immune microenvironment-mediated resistance	Reduced bone marrow natural killer cells, inhibitory effector-cell phenotypes, T-cell exhaustion, and downregulated tumor-cell CD38 transcription	Impaired immune-mediated killing and acquired resistance	Exploratory resistance features; not validated for routine treatment selection	(115)
Retreatment or within-class switching after anti-CD38 exposure	CD38 expression and antibody sensitivity may partially recover after a treatment-free interval	Retreatment may benefit selected patients, whereas evidence for within-class switching remains limited	Not established for routine use; consider prior response, treatment-free interval, and available alternatives	(122–125)

This table summarizes major safety, laboratory, and resistance issues discussed in the text and is intended to provide practical considerations rather than exhaustive management recommendations. ABO/RhD, ABO and RhD blood grouping; CD55/CD59, complement-inhibitory proteins; IgG-kappa, immunoglobulin G kappa; M-protein, monoclonal protein.

### 6.1 Safety considerations

The safety profile of anti-CD38 monoclonal antibodies is generally manageable, but proactive monitoring is required because treatment affects normal plasma cells and selected immune-effector populations. Administration-related reactions occur mainly during the first dose and decrease with subsequent administration. Subcutaneous delivery has reduced the administration burden of anti-CD38 therapy. Subcutaneous daratumumab has shown non-inferior efficacy and pharmacokinetic exposure compared with intravenous administration while reducing administration-related reactions and treatment time (28). More recently, the phase III IRAKLIA study similarly demonstrated non-inferior efficacy and pharmacokinetic exposure for subcutaneous isatuximab compared with intravenous isatuximab, with substantially fewer infusion-related reactions (27). These developments support subcutaneous administration as an important strategy for improving treatment convenience while maintaining clinical efficacy. Premedication and appropriate monitoring remain important during early dosing, particularly in older or frail patients.

Reduction of polyclonal immunoglobulins is a clinically observed consequence of CD38-directed depletion of normal antibody-secreting cells. This effect has also been documented outside multiple myeloma: daratumumab reduced immunoglobulin levels in patients with immune thrombocytopenia, and CM313 produced greater reductions in several immunoglobulin classes than placebo in a randomized study of systemic lupus erythematosus (36, 81). In nonmalignant or off-label settings, the clinical consequences of this immune depletion remain less well defined because prospective experience is limited and background immunosuppression differs from that in multiple myeloma.

In multiple myeloma, infection risk is multifactorial and should be interpreted at the regimen level. Randomized-trial evidence indicates that anti-CD38-containing regimens can increase the risk of infection, but disease-related immune dysfunction, prior therapy, corticosteroids, and other partner drugs also contribute (108, 109). Baseline assessment should therefore include infection history, comorbidities, vaccination status, and immunoglobulin levels. Vaccination, antiviral or other antimicrobial prophylaxis, and immunoglobulin replacement should be individualized according to the treatment regimen and patient-specific risk (116, 117).

Cytopenias are similarly multifactorial and regimen-dependent. Shared-backbone phase 3 trials have reported higher neutropenia rates in some anti-CD38-containing arms, indicating that addition of the antibody can contribute to hematologic toxicity, while myelosuppressive partner drugs, prior therapy, marrow reserve, and disease burden also remain important (47, 51, 109). Management should consider the entire treatment regimen, with blood count monitoring, supportive care, and adjustment of myelosuppressive partner drugs when appropriate rather than automatic discontinuation of anti-CD38 therapy.

### 6.2 Laboratory interference

Laboratory interference is a distinctive implementation issue with anti-CD38 monoclonal antibodies. Because red blood cells express low levels of CD38, daratumumab can bind reagent red cells and cause panreactivity in antibody screening and identification, with persistently positive indirect antiglobulin tests (110). To avoid transfusion delays, patients should undergo baseline ABO/RhD typing, antibody screening, and extended red cell phenotyping or genotyping when feasible before treatment. The transfusion service should also be informed of anti-CD38 exposure.

Dithiothreitol treatment of reagent red cells is the most widely used method to resolve daratumumab-related interference, but it denatures Kell antigens; therefore, Kell-negative red blood cells should generally be provided if the patient’s Kell phenotype is unknown (118). Isatuximab can also interfere with indirect antiglobulin testing, although its pattern may differ from daratumumab (111).

Anti-CD38 antibodies may also interfere with myeloma response assessment. Daratumumab is an immunoglobulin G kappa monoclonal antibody and may appear as a monoclonal band on serum protein electrophoresis or immunofixation electrophoresis, especially in patients with IgG-kappa myeloma (112, 119). Daratumumab-specific immunofixation assays and mass spectrometry-based methods can help distinguish therapeutic antibody from endogenous monoclonal protein (119–121). Standardized pre-treatment documentation and communication with laboratory services are therefore essential.

### 6.3 Response heterogeneity and resistance

Despite the substantial clinical benefit of anti-CD38 monoclonal antibodies in multiple myeloma, responses are heterogeneous and acquired resistance reflects interactions among target availability, complement activity, immune-effector function, prior treatment exposure, and the bone marrow immune microenvironment. Lower baseline CD38 expression has been associated with reduced response to daratumumab, and progression during therapy may be accompanied by CD38 downregulation and increased complement-inhibitory proteins such as CD55 and CD59 (113). Biallelic disruption of the CD38 locus has also been described as a mechanism of antigen escape in resistant disease (114).

Resistance is also shaped by the immune microenvironment. Acquired daratumumab resistance has been associated with reduced bone marrow natural killer cells, inhibitory effector-cell phenotypes, T-cell exhaustion, and downregulated tumor-cell CD38 transcription (115). These findings indicate that anti-CD38 efficacy depends on both tumor-intrinsic susceptibility and immune-effector competence.

Clinical evidence for anti-CD38 retreatment remains limited. In a retrospective study of 43 patients with daratumumab-refractory multiple myeloma, daratumumab-based retreatment in combination with other anti-myeloma agents produced a response rate of 49% and a median PFS of 7.97 months (124). Translational data also suggest that CD38 expression and ex vivo sensitivity to CD38 antibodies may recover after a treatment-free interval, with greater sensitivity observed after longer intervals from prior daratumumab exposure (122, 123).

Evidence for switching between anti-CD38 antibodies is also limited. In a phase 2 study of 32 heavily pretreated patients with daratumumab-refractory multiple myeloma, isatuximab monotherapy produced no objective responses, although the disease control rate was 37.5% and was higher in patients with a longer interval from prior daratumumab exposure (125). These findings do not establish within-class switching as a strategy to overcome anti-CD38 resistance.

## 7 Future directions and conclusions

The clinical development of anti-CD38 monoclonal antibodies is entering a new phase. In multiple myeloma and immunoglobulin light-chain amyloidosis, the central question is no longer whether CD38 targeting is effective, but how best to optimize its use across disease stages, treatment backbones, and patient subgroups. In multiple myeloma, key issues include sequencing after frontline anti-CD38 exposure (2), retreatment after prior exposure (122, 124), integration with other immune-based therapies, and MRD-guided treatment adaptation (60, 66). In AL amyloidosis, unresolved questions include the management of advanced cardiac involvement and the clinical relevance of high-risk clonal features (63, 64, 67).

Beyond established plasma cell disorders, the major challenge is to identify where CD38 targeting is biologically plausible, clinically relevant, and supported by sufficiently mature evidence. The most compelling emerging areas are those in which disease biology depends on pathogenic antibody-producing cells or humoral immune injury, including autoimmune cytopenias, hematopoietic stem cell transplantation-related antibody-mediated complications, and antibody-mediated rejection in transplantation (10, 29, 35, 83, 102). Expansion into broader autoimmune, renal, neurologic, or solid-organ transplant settings should be guided by disease-specific biology and prospective validation rather than by CD38 expression alone.

MRD status has important prognostic value in multiple myeloma and is increasingly being incorporated into prospective treatment-adaptation strategies (60, 66). In contrast, associations involving tumor-cell CD38 expression, complement-inhibitory proteins, biallelic CD38 disruption, and bone marrow immune features have been reported in translational or relatively small clinical studies and should be regarded as exploratory rather than as validated treatment-selection biomarkers (113–115, 123). In autoimmune and alloimmune diseases, candidate biomarkers such as autoantibody or donor-specific antibody kinetics and plasma-cell signatures also require prospective validation.

In conclusion, anti-CD38 monoclonal antibodies have an established therapeutic role in multiple myeloma and light-chain amyloidosis, whereas evidence in most other diseases remains preliminary. CD38 expression alone is insufficient to justify therapeutic use outside these settings. Future development should focus on diseases in which pathogenic plasma cells or antibody-producing cells have a central biological role and in which prospective studies can demonstrate clinically meaningful benefit with an acceptable safety profile.
